# Synthesis of nanohybrid consisting of taurine derived carbon dots and nanoceria for anticancer applications

**DOI:** 10.1016/j.toxrep.2024.101794

**Published:** 2024-10-30

**Authors:** Joydeep Das, Th. Abhishek Singh, R. Lalruatsangi, Parames C. Sil

**Affiliations:** a Department of Chemistry, School of Physical Sciences, Mizoram University, Aizawl, Mizoram 796004, India; b Project Assistant ll, Shoolini University, Solan, Himachal Pradesh, 173229, India; c Division of Molecular Medicine, Bose Institute, P-1/12, CIT Scheme VII M, Kolkata 700054, India

**Keywords:** Breast cancer, Carbon dots, Nanoceria, Reactive oxygen species, Taurine

## Abstract

Here, we first synthesized carbon dots (CDs) from taurine and then a nanohybrid with ceria (CeO_2_) nanoparticles using thermal decomposition method for checking their antineoplastic efficacy against human triple negative mammary carcinoma cells, MDA-MB-231. The in vitro study demonstrated significant dose-dependent antineoplastic activity of the nanohybrids within a range of concentration from 10 to 50 μg/mL after 48 h of treatment. The cellular morphology analysis clearly depicted substantial amount of cell death which seems to stem from increased intracellular reactive oxygen species (ROS) production. However, the maximum anticancer activity of the nanohybrid as compared to bare CDs and CeO_2_ is supposed to be due to the combined anticancer effect of both CDs and CeO_2_ (a well-established antitumor agent). Further we have performed molecular docking study to reveal the anticancer mechanism of CDs which exhibited high binding capacity towards several proapoptotic and antiapoptotic protein molecules. The binding affinity values were found to be – 8.7 kcal/mol, – 7.9 kcal/mol, – 9.6 kcal/mol, – 9.5 kcal/mol, – 12 kcal/mol and – 11.1 kcal/mol for BAD, BCl-2, p53, Caspase-8, Caspase-9 and Caspase-3 respectively. Taken together, our synthesized CDs-CeO_2_ nanohybrid could be thought as a potential anticarcinogenic option in the field of breast cancer therapeutics.

## Highlights

• A novel CeO_2_/CD nanohybrids were synthesized.
• CeO_2_/CD exhibited superior anticancer activity against MDA-MB-231 cells.
• CeO_2_/CD increased ROS production in MDA-MB-231 cells.
• CD showed stable interactions with apoptotic proteins.

## 1 Introduction

Despite of advances in recent onco-research and chemotherapeutic development, cancer prevails as a prevalent and deadly disease affecting nearly millions of people worldwide which is caused due to the uncontrolled growth of cells owing the potential to translocate into another body parts [1], [2], [3]. Although chemotherapy is accepted as the most effective choice for cancer treatment but sometimes it remains associated with poor treatment outcomes and side effects to the vital body organs. Currently, cancer nanotherapy or nanoscale delivery of antineoplastics agents are in increasing demand due to their high potential for precise specificity and multifunctionality [4], [5].

Nanoparticles (NPs) exhibit superior chemical, thermal, optical, electronic, and magnetic properties as compared to their bulk materials and due to their large surface area to volume ratio. Besides, they are increasingly gaining interest among biological researchers due to their numerous therapeutic

and theranostic applications [6], [7], [8]. Nanoparticles are also been used in the field of cancer detection, diagnosis and therapy. This new emerging field of nano-oncology shows superior applications including targeted drug delivery, biomolecular study of cancer biomarkers, and in vivo tumor imaging. Moreover, nanoparticles are also useful for cancer immunotherapy [9] Nanoparticles can also be used as photothermal or photodynamic therapeutic agents for cancer treatment [10]. Recently, it has been found that carbon dots (CDs) obtained from various natural or green resources offer significant biomedical applications [11], [12]. Additionally, potential biomedical applications of amino acid derived CDs are also well documented [13], [14], [15], [16], [17]. For instance, taurine is a Sulphur containing non-proteinogenic amino acid which occurs naturally inside the human body. It can be found in egg, meat, milk, fish and available as dietary supplement as well [18], [19], [20]. It is reported that taurine can be used in several biological applications such as, improve vision, brain and heart function; has protective role against diabetes, high level of fat in blood. Recently, antineoplastic role of taurine has also been highlighted in several types of cancer including colorectal carcinoma, ulcerative colitis, lung carcinoma etc [21], [22], [23], [24], [25], [26], [27]. Thus, in current study, we have nano-formulized taurine by synthesizing novel taurine-derived CDs. Besides, nanocomposites or nanohybrids containing CDs and multiple other nano-metal oxides are reported to show enhanced anticancer activity [11], [28], [29], [30], [31]. In this context, nanoceria (CeO_2_) is gaining huge interest of nanobiotechnologists due to their unique physicochemical properties and non-toxic profile along with profound antioxidant, antibacterial, anticancer and antidiabetic properties [32].

Besides, nanoceria, have a unique redox activity such as switching between Ce^3+^ and Ce^4+^ oxidation states, which makes them one of the most promising therapeutic agents for the treatment of cancer via the formation of reactive oxygen species [33]. Nano-CeO_2_ exhibits pH-dependent pro-oxidant or antioxidant activities. Under physiological condition (in normal cells) it behaves as antioxidant, whereas under mildly acidic condition (such as in cancer cells), it behaves as prooxidant and kill cancer cells via oxidative stress [32].

In our present study, we have focused upon the synthesis of a novel anticancer nanohybrid (CDs-CeO_2_) combining both the anticancer properties of taurine-derived CDs and nanoceria. We have also assessed its anticancer properties against human mammary cancer cells MDA-MB-231 in vitro accompanying with molecular docking studies to reveal the anticancer mechanisms of the as-synthesized novel CDs. Thus, this study postulates that the CDs-CeO_2_ nanohybrid would be a highly efficient anticancer agent owing the inherent anticancer potential of both nanoceria and CDs.

## 2 Material and methods

### 2.1 Regents

Taurine was procured from Loba Chemie Pvt. Ltd. (Mumbai, India), while the NaOH (98 %) was procured from RFCL Limited (RANKEM) (Delhi, India). Ammonium ceric nitrate (98 %), disodium dihydrogen phosphate dihydrate and potassium dihydrogen phosphate were obtained from Central Drug House (P) Ltd. (New Delhi, India). MTT was procured from Sisco Research Laboratory (Mumbai, India). Cell culture media (DMEM) and FBS were obtained from Hi-Media (Mumbai, India).

### 2.2 Preparation of CDs, nano-CeO_2_, and CDs-CeO_2_ nanohybrids

The detailed preparation methods of CDs, nano-CeO_2_, and CDs-CeO_2_ nanocomposites and their detailed characterization are well documented in our previous publications [14], [15]. In brief, CDs and nano-CeO_2_ were synthesized via heating taurine and cerium hydroxide separately within muffle furnace at 350 °C for 15 min. Similarly, CDs-CeO_2_ nanohybrids were prepared from the mixture of taurine and cerium hydroxide by heating at 350 °C for 10 min.

### 2.3 Cell culture

In present study, human triple negative epithelial mammary cancer (TNBC) cells MDA-MB-231 (acquired from NCCS, Pune, India) were routinely cultured within DMEM media added with 10 % FBS in a humified incubator containing 5 % CO_2_ at 37 °C temperature. To prevent probable bacterial and fungal infection media was also added with 1 × 10^5^ IU/L penicillin and 0.1 g/L streptomycin. Cells were allowed to be 70 % confluent (having seeding concentration of 5 × 10^5^ cells per well) prior to further experiments.

### 2.4 Determination of cytotoxic profile

The detailed experimental procedures are already documented in our earlier publications [34]. Briefly, MDA-MB-231 cells were treated with CDs, nano-CeO_2_, and nanohybrid CDs-CeO_2_ at pre-decided concentrations from 10 μg/mL to 50 μg/mL for 48 h and the cell viability was investigated using a standard MTT assay through spectroscopic analysis at 570 nm.

### 2.5 Determination of reactive oxygen species (ROS)

Determination of ROS in nano-treated MDA-MB-231 cells, has been made following the procedure described elsewhere [35]. In short, 25 μg/mL of each of CDs, nano-CeO_2_ as well as nanohybrid CDs-CeO_2_ was added to separate wells of treatment group and were incubated with for 48 hrs followed by scrapping and centrifugation. Collected cell-pellets were PBS-washed and resuspended in cell wash followed by incubation with 2 μM H_2_DCFDA (for 20 min in dark at 37 °C). Finally, samples were examined for intracellular ROS in term of proportionate green fluorescence by flow cytometer at 520 nm.

### 2.6 Molecular docking study

CB-DOCK2 web server has been utilized to perform the molecular docking study for checking the interaction of CDs and several pro- and anti-apoptotic protein molecules (Table 1). The probable structure of CDs has been drawn using Gaussview 5.0 software and used for blind docking by selecting five cavities. Among the cavities, the one showing the highest binding affinity has been chosen and BIOVIA Discovery Studio has been utilized for the visualization of the 2D interaction plots and the different binding poses of CDs-protein complexes.

**Table 1:** Molecular docking results of drugs with proteins.

**Compound**	**Proteins**	**PDB ID**	**Binding sites**	**Binding affinity (kcal/mol)**
Carbon dots	BCl2	6VO4	TYR(8), ARG (135), GLY(138), TRP(140), GLU(141), and ASN(142)	− 7.9
	BAD	1G5J	PHE(135), ARG(136), VAL(139), TYR(177), PRO(184) and TRP(185)	− 8.7
	p53	4HG7	ARG(10), GLN(23), TYR(92), ARG(203), SERE(228), VAL(229) and ASN(232)	− 9.6
	Caspase-9	2AR9	GLU(143), GLY(147), ALA(149), ASP(150), TYR(153), ARG(408), ILE(154), ASP(228), GLY(276), GLY(277), LYS(278) and LYS(409)	− 12.0
	Caspase-8	3KJN	ARG(260), CYS(360), TYR(412), ARG(413) and ASP(455)	− 9.5
	Caspase-3	3KJF	ARG(64), HIS(121), GLN(161), CYS(163), SER(205), TRP(206), ARG(207), ASP(253) and PHE(256)	− 11.1

### 2.7 Statistical analysis

Experiments have been repeated thrice and the data have been plotted in terms of mean ± SD values. Statistical analyses were performed with OriginPro 8.0 software by employing one way analysis of variance (ANOVA) with Tukey test where *P*-value < 0.05 has been considered to determine statistically significant difference.

## 3 Results and discussion

### 3.1 Analysis of in vitro cytotoxicity of CDs, CeO_2_ and CDs-CeO_2_ nanocomposites

**Fig. 1:**
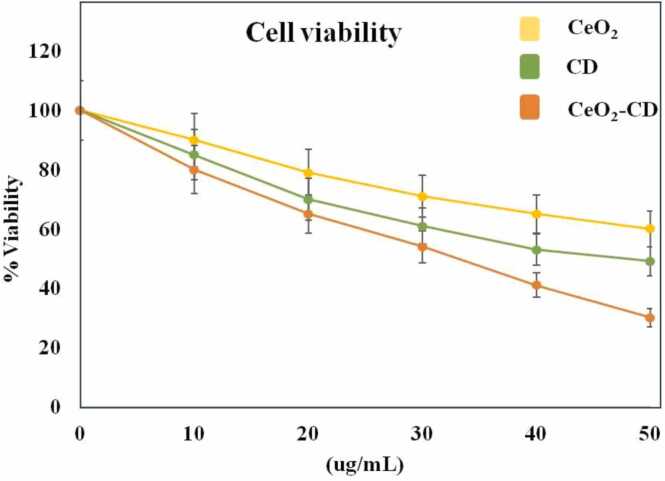
In vitro cytotoxicity (cell viability) measurement via MTT assay of MDA-MB-231 cells after treating with CDs, nano-CeO_2_ and CDs-CeO_2_ nanohybrids. All the data are representative of three independent experiments and the values are expressed as mean ± SD.

CDs derived from several natural sources are reported to exhibit profound anticancer activity [12], [17], [36], [37]. Similarly, CeO_2_ nanoparticles are also well known for their cancer cell specific cytotoxicity [32], [38], [39], [40]. These previous reports prompted us to check the anticancer potential of the novel CDs-CeO_2_ nanohybrids against human triple negative mammary carcinoma cells, MDA-MB-231. In this study, carbon dots (CDs), CeO₂ nanoparticles and CDs-CeO₂ nanohybrids were fabricated by heating the taurine and cerium hydroxide in a muffle furnace at 350 °C for 10–15 min. In this nanocomposite the attractive forces acting between CDs and CeO₂ nanoparticles are ionic interaction between Ce⁴⁺/Ce⁴⁺ ions and the sulphate groups of the CDs, as well as the lone pair on nitrogen. Additionally, hydrogen bonding between the N—H bond of CD and oxygen atoms in CeO₂ may also contribute to this interaction. The synthesized CDs, CeO_2_ as well as CDs-CeO_2_ nanohybrids have already been thoroughly characterized by our research group [14], [15]. Dose-dependent cytotoxic effects of CDs and nano-CeO_2_ were visible against MDA-MB-231 cells when treated for 48 hrs (Fig. 1) with respective IC_50_ doses, i.e., 44.11 μg/mL and 59 μg/mL. Likewise, CDs-CeO_2_ nanohybrid exhibited dose dependency against MDA-MB-231 cells with highest cytotoxicity among treated groups (IC_50_ 33.54 μg/mL) over a range of 10–50 µg/mL.

### 3.2 Morphological assessment of MDA-MB-231 cells following treatment with CDs, CeO_2_ and CDs-CeO_2_ nanocomposites

**Fig. 2:**
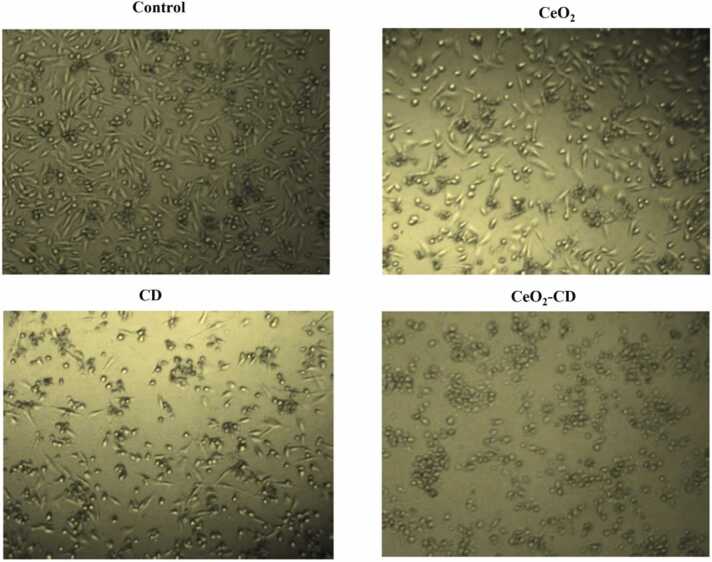
Cellular morphology of MDA-MB-231 cells after treating with CDs, nano-CeO_2_ and CDs-CeO_2_ nanohybrids. Images were taken at 40 × magnification.

Morphological assessment of MDA-MB-231 cells has also been carried out after treatment with 25 μg/mL of CDs, nano-CeO_2_ as well as CDs-CeO_2_ nanohybrid separately for 48 h to correlate with the cell viability results. The bright field optical microscopic images in Fig. 2 showed that both CDs and nano-CeO_2_ caused significant morphological alterations in MDA-MB-231 cells including cellular shrinkage, clumping, no lamellar expansions, loss of membrane integrity, cytoplasmic condensation, and formation of apoptotic bodies. However, treatment with nanohybrid at the same concentration again caused the maximum cytotoxic effect as almost all the cells were found dead.

### 3.3 ROS generation

**Fig. 3:**
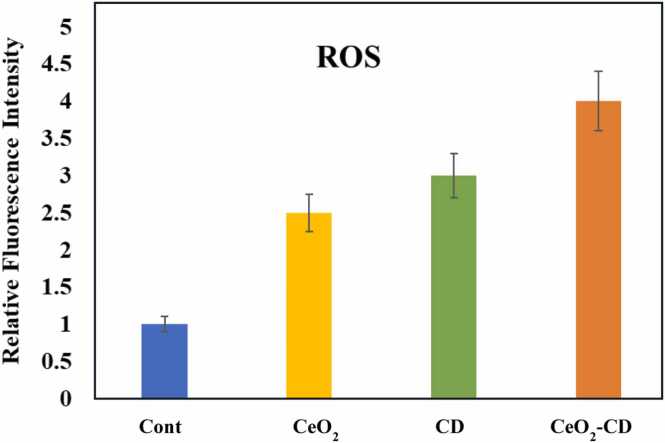
In vitro effect of the synthesized nanomaterials upon intracellular reactive oxygen species (ROS) formation depletion in MDA-MB-231 cells. Effect of 25 μg/mL CDs, CeO_2_ and CDs-CeO_2_ nanohybrid-treatment upon ROS production using DCFH-DA staining in MDA-MB-231 cells. All the data are representative of three independent experiments and the values are expressed as mean ± SD.

Cytotoxicity or cell death due to nanoparticle exposure often results from oxidative stress [41]. ROS is playing a key role to control the biological functions in both normal and diseased cells. In physiological conditions, ROS activate several cell signaling pathways responsible for growth and development. On the other hand, under stressed conditions, ROS trigger pathological effects as well as cell death via activating either apoptotic or necrotic pathway [42], [43], [44], [45], [46], [47]. Besides, CeO_2_-induced ROS production and apoptotic cell death in cancer cells has already been reported [32], [38], [39], [40]. Therefore, we have analyzed the intracellular ROS burden following the exposure of all the synthesized nanomaterials. It has been found that at a dose of 25 µg/mL, nano-CeO_2_ increased the ROS level almost 2.5 times higher than the untreated control. However, at the same concentration, CDs has been found to be slightly higher potent (3 times higher than untreated cells) than nano-CeO_2_ with maximum rise in ROS level caused by CDs-CeO_2_ nanohybrid treatment (4.5 times higher than untreated cells) (Fig. 3). Thus, rise in ROS level may contribute to the progression of cell death with hybridization of nano-CeO_2_ and CDs.

The ROS formation ability of CDs can be attributed to the different functional groups attached to their surface [48]. The presence of oxygen-containing functional groups, such as carbonyls, hydroxyls, and carboxylic groups are essential for the ROS formation. It has been observed that removal of these functional groups significantly reduces their ability to generate ROS. The structural defects present in the sp² carbon domain of CDs improve their electronic features, thereby enabling effective charge separation and ROS production upon light irradiation [49], [50], [51]. Additionally, following ROS production CDs can also trigger apoptotic cell death pathway via up-regulation of Bax, down-regulation of Bcl-2 and activation of Caspase 3 [52].

### 3.4 Evaluation of molecular mechanism involved in anticancer property of CeO_2_-CDs nanohybrids

Apoptotic cell death may occur mostly via mitochondria dependent (intrinsic) and mitochondria independent pathways (extrinsic) [53], [54], [55]. In the intrinsic pathway, the activation of pro-apoptotic BH3-only proteins, such as Bax and Bak, suppress the anti-apoptotic proteins Bcl-2, Bcl-xL, and Mcl-1, resulting in disruption of mitochondrial outer membrane permeability. This allows proteins that are usually limited to the intermembrane gap to leak into the cytosol, including apopotogenic factors such as cytochrome c. Cytochrome c then attaches to Apaf-1 (apoptosis protease activating factor-1), creating the apoptosome complex, which recruits pro-Caspase-9, which further activate Caspases-3, -6, and -7, resulting in apoptotic cell death [56], [57], [58], [59]. On the other hand, apoptosis can also be triggered by the activation of death receptors, such as TNFR1 and FasR which transmit the death signal from cell surface to inside the cell. These FasL/FasR and TNF-α/TNFR1 ligands-death receptors activation further activate Caspase-8 and promote cell death via extrinsic pathway [60]. Since increase in the ROS level often triggers both intrinsic and extrinsic apoptotic cell death pathways [61], we therefore performed the molecular docking study to check the binding affinity of the novel CDs towards several apoptotic proteins such as BAD, BCl-2 (PDP ID: 6VO4), p53 (PDP ID: 4HG7), Caspase-8 (PDP ID: 3KJN), Caspase-9 (PDP ID: 2AR9) and Caspase-3 (PDP ID: 3KJF).

**Fig. 4:**
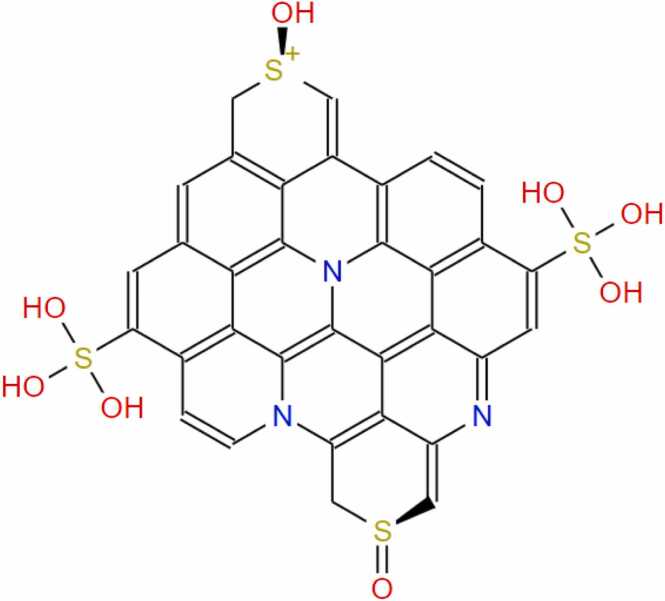
Proposed structure of the synthesized CDs which is used for molecular docking study.

**Fig. 5:**
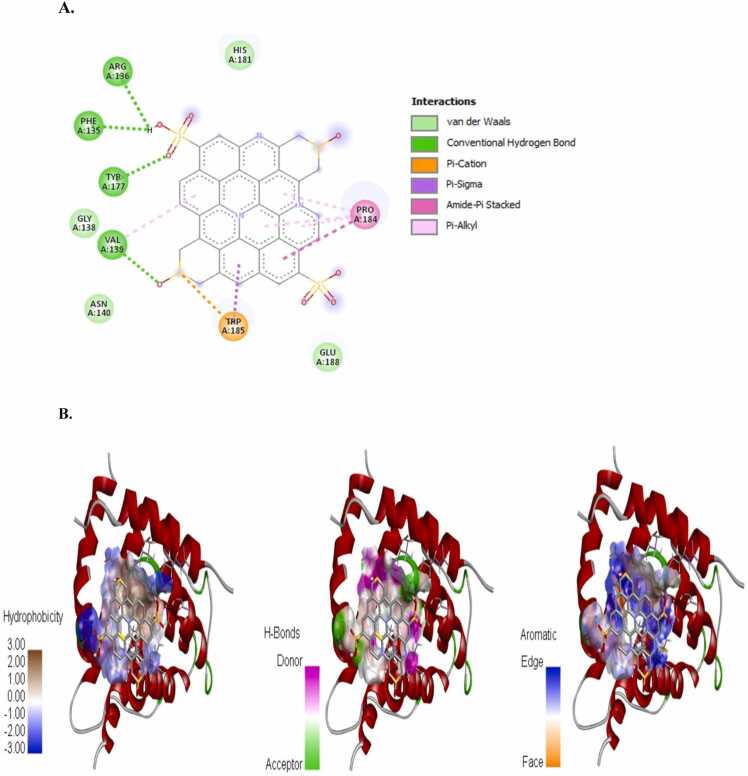
Molecular docking study of CDs and BAD protein. (a) Interaction plot inside the binding pocket and (b) favorable binding poses showing hydrophobic interactions, hydrogen bonded interactions and aromatic (pi) interactions.

**Fig. 6:**
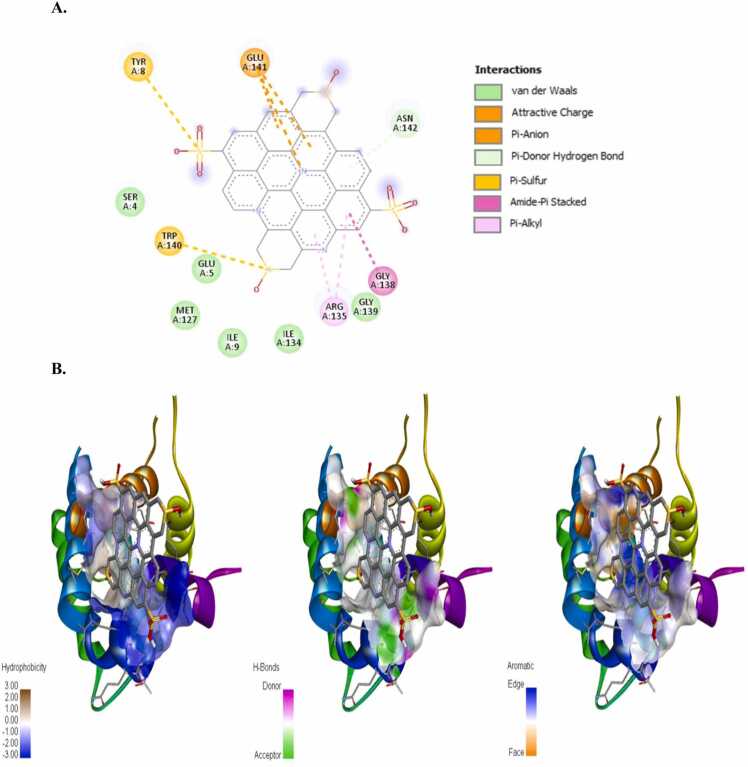
Molecular docking study of CDs and BCl_2_ protein. (a) Interaction plot inside the binding pocket and (b) favorable binding poses showing hydrophobic interactions, hydrogen bonded interactions and aromatic (pi) interactions.

**Fig. 7:**
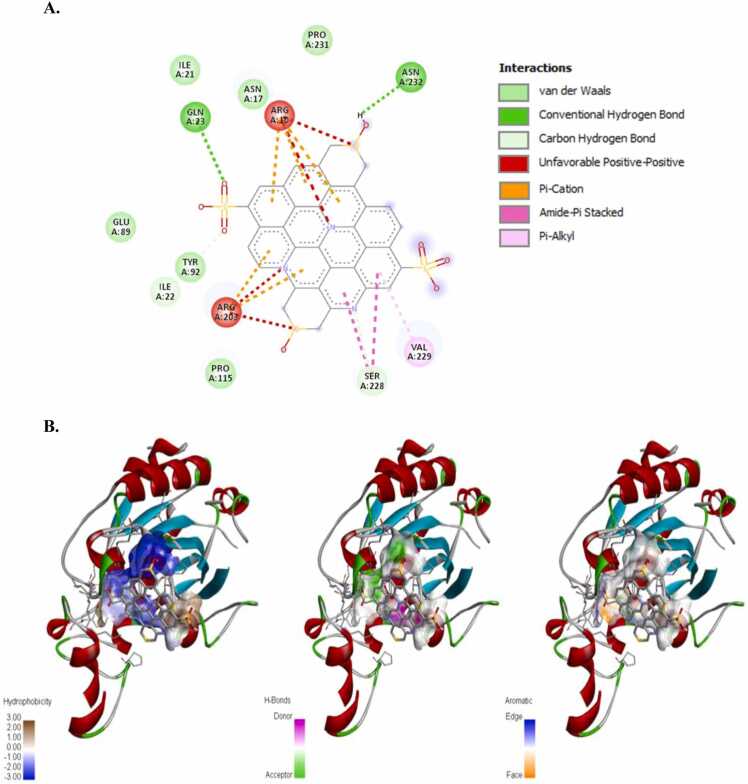
Molecular docking study of CDs and p53 protein. (a) Interaction plot inside the binding pocket and (b) favorable binding poses showing hydrophobic interactions, hydrogen bonded interactions and aromatic (pi) interactions.

**Fig. 8:**
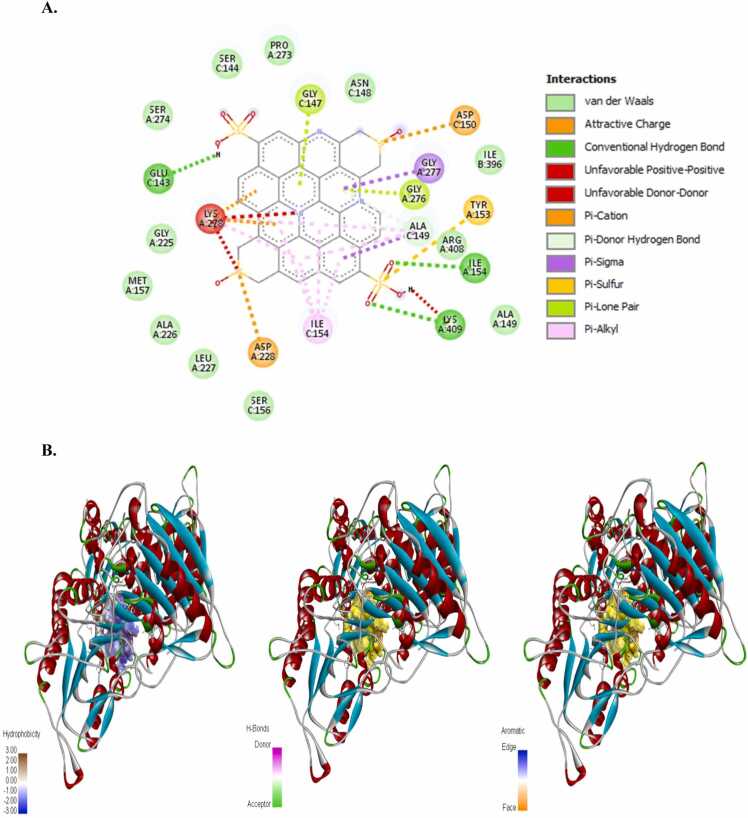
Molecular docking study of CDs and Caspase-9 protein. (a) Interaction plot inside the binding pocket and (b) favorable binding poses showing hydrophobic interactions, hydrogen bonded interactions and aromatic (pi) interactions.

**Fig. 9:**
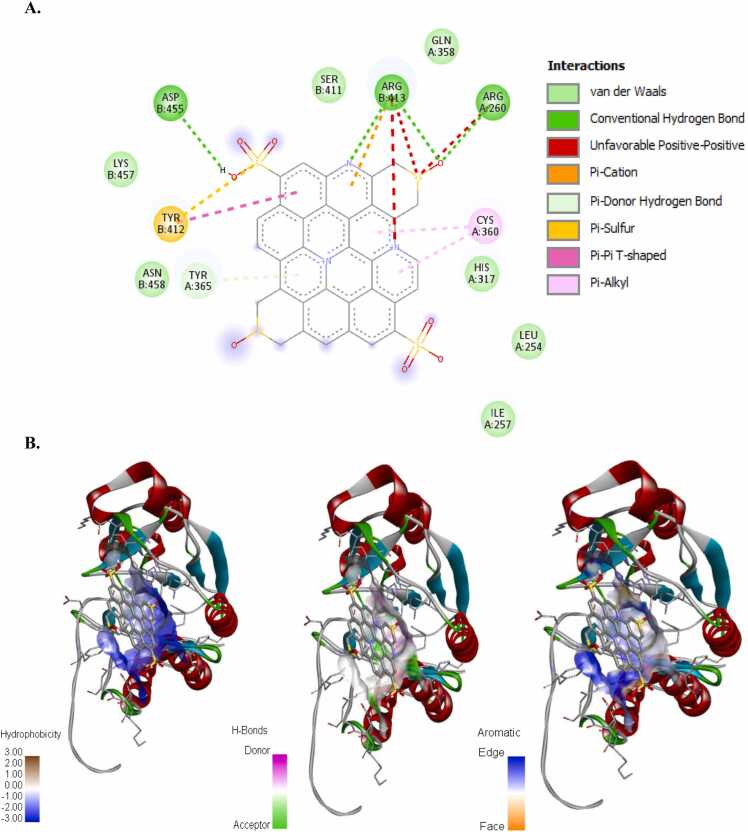
Molecular docking study of CDs and Caspase-8 protein. (a) Interaction plot inside the binding pocket and (b) favorable binding poses showing hydrophobic interactions, hydrogen bonded interactions and aromatic (pi) interactions.

**Fig. 10:**
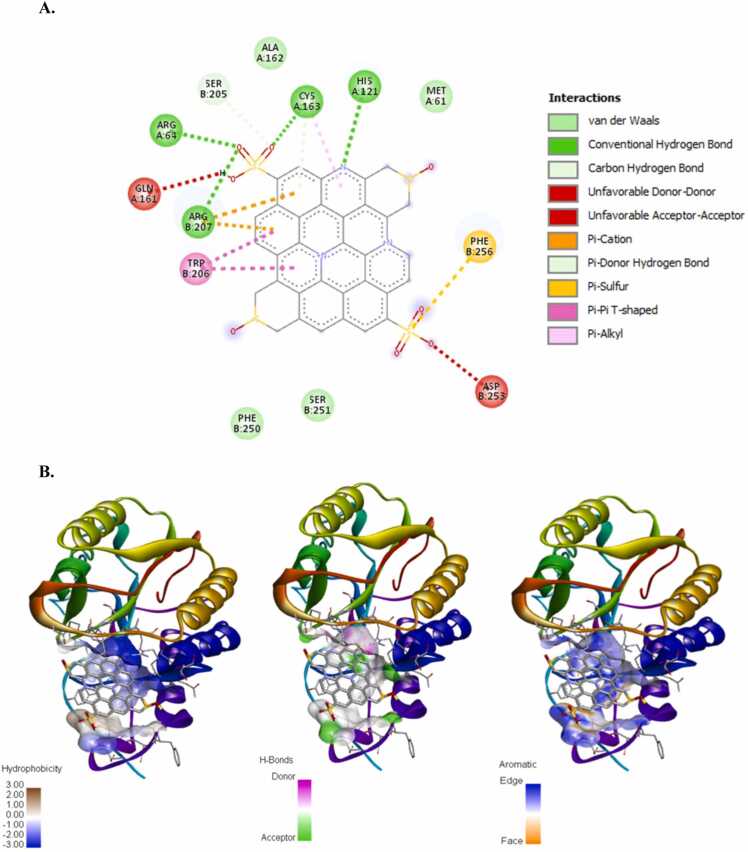
Molecular docking study of CDs and Caspase-3 protein. (a) Interaction plot inside the binding pocket and (b) favorable binding poses showing hydrophobic interactions, hydrogen bonded interactions and aromatic (pi) interactions.

The incidence of apoptosis in cancer cells due to CeO_2_ exposure has already been reported [32], [38], [39], [40], therefore, we did not conduct any molecular docking study with nanoceria. The structure of the synthesized CDs has been shown in Fig. 4. The CD-receptor non-bonded interaction plots were shown in Fig. 5, Fig. 6, Fig. 7, Fig. 8, Fig. 9, Fig. 10, whereas the binding affinities and interactive amino acids were listed in Table 1. CDs exhibited binding affinity values of – 8.7 kcal/mol, – 7.9 kcal/mol, – 9.6 kcal/mol, – 9.5 kcal/mol, – 12 kcal/mol and – 11.1 kcal/mol for BAD, BCl-2, p53, Caspase-8, Caspase-9 and Caspase-3 respectively. These high binding values are indicating strong and stable binding interactions of CDs with the selected protein molecules. For BAD proteins, the important non-bonded interaction types were pi-Alkyl, amide-pi stacking, pi-Sigma, pi-Cation and hydrogen bond interactions in nature (Fig. 5). For BCl-2 proteins, the important non-bonded interaction types were pi-Sulfur, pi-Alkyl, amide-pi stacking, pi-Anion and electrostatic interactions in nature (Fig. 6). Similarly, for p53 proteins, the interaction types were hydrogen bond, pi-Alkyl, amide-pi stacking, pi-Cation and unfavorable positive-positive interactions (Fig. 7). On the other hand, for Caspase-9, the interaction types were hydrogen bond, pi-Alkyl, pi-lone pair, pi-Cation, pi-Sulfur, pi-Sigma and unfavorable positive-positive as well as Donor-Donor interactions (Fig. 8). For, Caspase-8, the interaction types were hydrogen bond, pi-Alkyl, pi-pi stacking, pi-Sulfur, pi-Cation and unfavorable positive-positive interactions (Fig. 9). Lastly, for Caspase-3, the interaction types were hydrogen bond, pi-Alkyl, pi-pi stacking, pi-Sulfur, pi-Cation and unfavorable Donor-Donor as well as Acceptor-Acceptor interactions (Fig. 10). This molecular docking study clearly indicates that the novel CDs may form stable complexes with all the tested apoptotic molecules, thereby affecting the survival of human triple negative mammary carcinoma cells, MDA-MB-231.

## 4 Conclusion

**Fig. 11:**
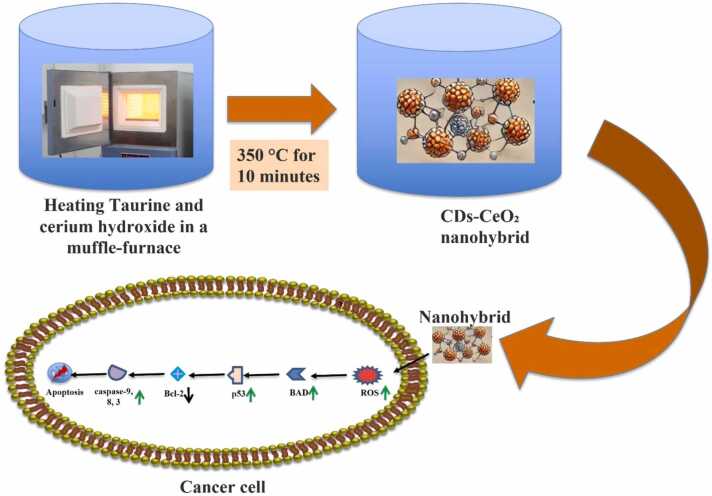
Schematic diagram of CDs-CeO_2_ nanohybrid synthesis and its anticancer activity.

Here, we have prepared a nanohybrid consisting of both CDs (from taurine) and CeO_2_ at 0.4 wt% CDs and checked their anticancer activities against human triple negative mammary carcinoma cell MDA-MB-231. Schematic diagram of the synthesis of nanohybrid, CD-CeO_2_ and its anticancer activity is presented in Fig. 11. The major attractive forces acting between CeO_2_ and CDs in the nanohybrid are ionic and hydrogen bonding interactions. The nanohybrid, CD-CeO_2_ exhibited potent cytotoxicity against MDA-MB-231 cells. Additionally, CD-CeO_2_ nanohybrids are found to induce intracellular ROS burst in MDA-MB-231 cells. The CDs are shown to exhibit high binding affinity with several key anti- and pro-apoptotic protein molecules such as, BAD, Bcl-2, p53, Caspase 9, 8 and 3. These binding interactions include various non-bonded interactions like hydrogen bonds, pi-Sulfur, pi-Alkyl, amide-pi stacking, pi-pi stacking, pi-lone pair, pi-Cation, pi-Anion and electrostatic. The formation of stable complexes between carbon dots (CDs) and several apoptotic proteins suggests apoptotic cell death pathway. All these results clearly highlighted the role of CD-CeO_2_ nanohybrids as an efficient chemotherapeutic agent.

## CRediT authorship contribution statement

**Joydeep Das:** Writing – review & editing, Writing – original draft, Visualization, Validation, Supervision, Software, Resources, Project administration, Methodology, Investigation, Funding acquisition, Formal analysis, Data curation, Conceptualization. **Abhishek Singh:** Validation, Software, Methodology, Investigation, Formal analysis, Data curation, Conceptualization, Writing – review & editing. **Parames Sil:** Writing – review & editing, Writing – original draft, Visualization, Validation, Supervision, Software, Project administration, Investigation, Conceptualization. **R. Lalruatsangi:** Data curation, Formal analysis, Investigation, Validation, Visualization.

## Declaration of Competing Interest

The authors declare that they have no known competing financial interests or personal relationships that could have appeared to influence the work reported in this paper.

## Data Availability

Data will be made available on request.
